# Larger neural responses produce BOLD signals that begin earlier in time

**DOI:** 10.3389/fnins.2014.00159

**Published:** 2014-06-12

**Authors:** Serena K. Thompson, Stephen A. Engel, Cheryl A. Olman

**Affiliations:** ^1^School of Medicine, University of MinnesotaMinneapolis, MN, USA; ^2^Department of Psychology, University of MinnesotaMinneapolis, MN, USA

**Keywords:** functional MRI, hemodynamics, visual cortex, linearity, causal modeling

## Abstract

Functional MRI analyses commonly rely on the assumption that the temporal dynamics of hemodynamic response functions (HRFs) are independent of the amplitude of the neural signals that give rise to them. The validity of this assumption is particularly important for techniques that use fMRI to resolve sub-second timing distinctions between responses, in order to make inferences about the ordering of neural processes. Whether or not the detailed shape of the HRF is independent of neural response amplitude remains an open question, however. We performed experiments in which we measured responses in primary visual cortex (V1) to large, contrast-reversing checkerboards at a range of contrast levels, which should produce varying amounts of neural activity. Ten subjects (ages 22–52) were studied in each of two experiments using 3 Tesla scanners. We used rapid, 250 ms, temporal sampling (repetition time, or TR) and both short and long inter-stimulus interval (ISI) stimulus presentations. We tested for a systematic relationship between the onset of the HRF and its amplitude across conditions, and found a strong negative correlation between the two measures when stimuli were separated in time (long- and medium-ISI experiments, but not the short-ISI experiment). Thus, stimuli that produce larger neural responses, as indexed by HRF amplitude, also produced HRFs with shorter onsets. The relationship between amplitude and latency was strongest in voxels with lowest mean-normalized variance (i.e., parenchymal voxels). The onset differences observed in the longer-ISI experiments are likely attributable to mechanisms of neurovascular coupling, since they are substantially larger than reported differences in the onset of action potentials in V1 as a function of response amplitude.

## Introduction

Most analyses of neuroimaging data assume a fixed shape for the measured response. In BOLD fMRI, for example, standard analyses measure neural activity by regressing data onto a cannonical hemodynamic response function (HRF); larger responses are assumed to be scaled copies of smaller ones. While scaling holds at least roughly (e.g., Boynton et al., 1996; Janz et al., 2001; Olman et al., 2004; Heckman et al., 2007; Li et al., 2008) it remains to be investigated in fine detail. In particular, it is unknown whether the timing of fMRI responses changes as neural activity increases. Testing this is important not only for standard analyses, but also for analyses that may attempt to use timing differences to infer order of operation, or causal relationships among regions of neural activity in the brain (Smith et al., 2011).

The current study was designed to test whether there is a relationship between the onset of a stimulus-evoked fMRI BOLD response and the amplitude of neural activity, as measured by the average magnitude of the BOLD response across voxels. We used high temporal resolution (*TR* = 250 ms) fMRI at 3 Tesla, and an event-related (ER) experimental design to allow detailed, accurate characterization of the hemodynamic response. Onset latency has previously been shown to vary across voxels, due to hemodynamic factors (Lee et al., 1995; de Zwart et al., 2005). Since we are primarily concerned with the relationship of onset with neural activity, rather than hemodynamics, we pooled responses across all active voxels prior to measuring onset.

## Materials and methods

**Figure 1:**
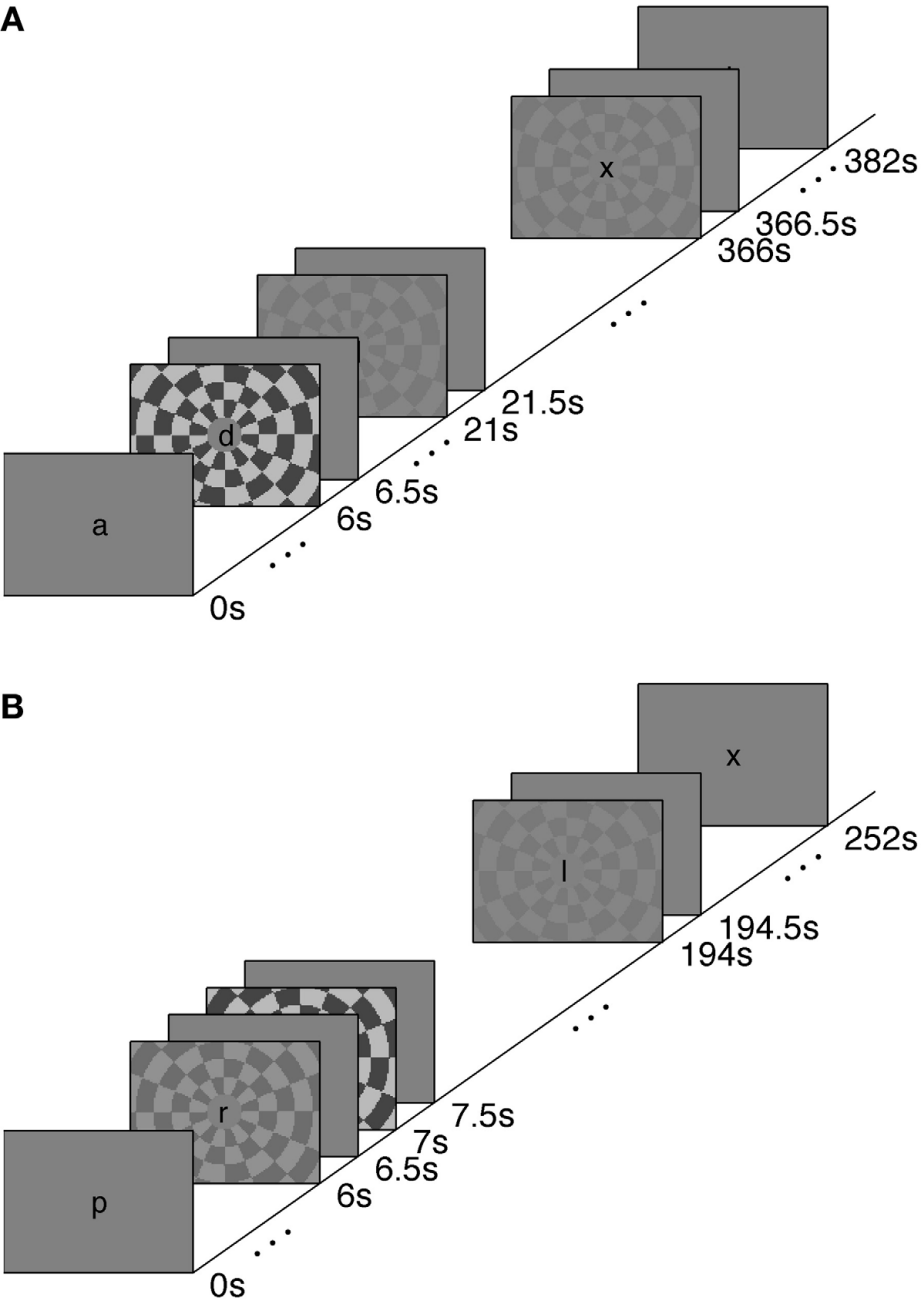
**Stimulus paradigms for Experiments 1 (A) and 2 (B)**. See text for details.

We measured the timing of visual, stimulus-driven, voxel average BOLD HRFs using a rapid-TR scanning protocol. We used stimuli at a range of contrasts—2.5, 5, 25, and 90%—to generate varying amounts of neural activity. The HRFs were elicited with a polarity-reversing achromatic checkerboard presented in ER designs. A long inter-stimulus interval (long-ISI) experiment was used to measure HRFs from neural activity produced by two stimulus contrasts; the HRFs were not affected by temporal overlap or the deconvolution necessary to produce event-triggered average responses. A short-ISI experiment allowed us to collect responses to four different contrast levels while keeping the total scan time approximately equal to the long-ISI experiment. Experimental design and stimuli are illustrated in Figure 1.

### Participants

Sixteen experienced observers [9 female, 7 male, ages 22–52 (mean 29)] participated in this study after providing written informed consent. Seven participants were aware of the experimental aims; three are authors. Four subjects (ages 25, 26, 35, and 43) participated in both the short- and the long-ISI experiments. An additional six subjects (ages 25, 25, 27, 30, 39, and 52) participated in the long-ISI experiment but not the short-ISI experiment; six other subjects (ages 22, 22, 24, 27, 32, and 39) participated in the short- but not long-ISI experiment. All participants had normal or corrected-to-normal visual acuity. Participation was voluntary and in compliance with the University of Minnesota Institutional Review Board guidelines.

### Stimuli and scanning paradigm

Visual stimuli were created using Matlab and the Psychophysics Toolbox extensions (Brainard, 1997; Pelli, 1997). Stimuli for the first four short-ISI and long-ISI scanning sessions (first four subjects in each experiment) were presented on a NEC 2190UXi monitor with a resolution of 1024 × 768 pixels and a refresh rate of 60 Hz. The luminance of the monitor was calibrated using a Photo Research PR-655 photometer. The monitor had a mean luminance of 110 cd/m^2^. The monitor was mounted to the back wall of the scanning suite; subjects viewed the monitor through a mirror mounted to the top of the head coil. Stimuli for the remaining six subjects in each experiment were presented on projection systems composed of a Sony projector fit with a custom lens made by Navitar, projecting the stimulus onto a screen behind the subjects' heads in the bore of the scanner. Two of the last six long-ISI and four of the last six short-ISI subjects were scanned in the Siemens Trio system, where the mean luminance of the projector was 120 cd/m^2^. Four of the last six subjects in the long-ISI and two of the six subjects in the short-ISI experiment were scanned in a Siemens Skyra system (details below) where the mean luminance of the projector was 640 cd/m^2^.

Data for three types of scans were collected: one block-design checkerboard used for region of interest (ROI) localization and two types of ER scan to collect timing data (short and long ISI). To elicit the HRF, all three types of scans employed checkerboard stimuli that reversed at a rate of 4 Hz extending across 12° of visual angle in the horizontal direction, and 9° in the vertical direction for the LCD display, 24° by 18° for the projection systems. All three scans also used a rapid serial visual presentation (RSVP) task to encourage participants to maintain fixation at the center of the screen. Letters were presented at the center of the screen at a rate of 4 Hz and subjects were instructed to press a button every time they saw the letter “x,” which occurred with a 10% probability.

**Figure 2:**
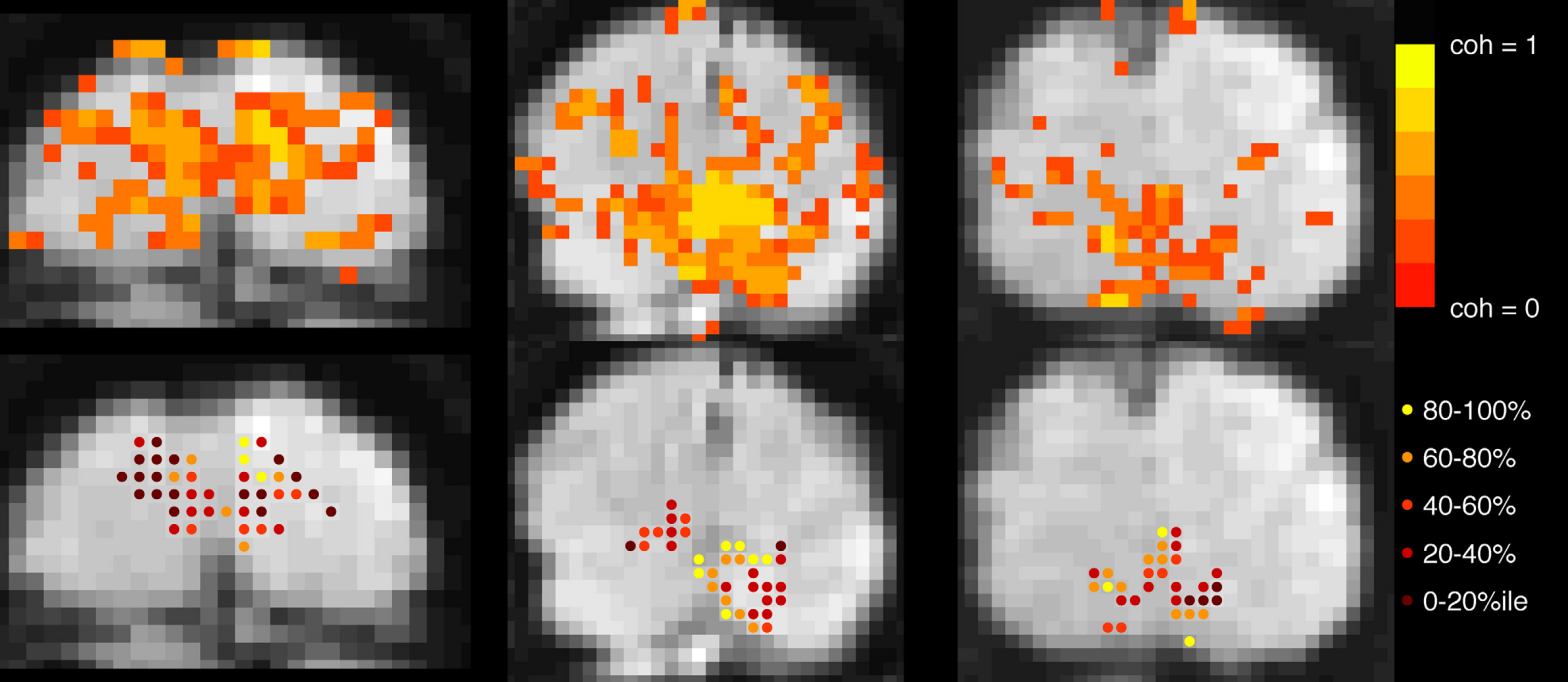
**Voxel selection. Top row**: Voxels with coherence > 0.30 during localizer scan are shown in color overlay. Gray-scale images are motion- and distortion-compensated EPI data from successive slices in the Trio scanner (left and right images) and, Skyra (middle image). **Bottom row**: Each active voxel in V1 is marked, with color indicating to which quintile it belonged after event-related data were assessed for mean-normalized variance. Voxels in the top quintile (yellow, 80–100%) were discarded before the main analysis to minimize contamination by sagittal sinus and poor CNR due to edge effects.

In both experiments, block-design scans were used to localize the cortical region corresponding to the retinotopic location of the checkerboard (Figure 2). Each 24-s block contained 12 s of contrast-reversing, 90% contrast checkerboard alternating with 12 s of a blank, gray screen. Each scan contained 10.5 blocks.

Experiment 1 was performed to measure the characteristics of the hemodynamic response stimulated from rest, or from a nearly relaxed state. Accordingly, we used an ISI of 15.5 s. Because the long-ISI ER design requires considerable time per trial, we limited this experiment to two contrasts (5 and 90%, ~7 min per scan with 9 repetitions of each stimulus contrast per scan). Trials contained a stimulus for 500 ms, followed by 15.5 s of rest. Each scan contained 24 events (6 blank, 9 5% contrast, 9 90% contrast, for 1700 TRs in 7 min, 5 s). Each subject participated in a single scanning session containing 4–6 long-ISI ER scans and 1 block-design localizer.

Experiment 2 used a shorter ISI (1 s), in order to collect the maximal number of stimulus responses with minimal subject fatigue. We measured responses to 2.5, 5, 25, and 90% contrast for 4–8 scans. Each trial contained a 500 ms stimulus followed by a blank screen for 500 ms (ISI of 1 s). Each scan contained 248 events (48 blank, 50 each of 2.5, 5, 25, and 90% contrast) for a total of 1008 TRs in 4 min, 12 s. Each subject participated in a single scanning session containing 1 block-design localizer scan and five or six short-ISI scans.

In both short- and long-ISI experiments, the order of conditions within a scan was counterbalanced using an m-sequence, an ordering of conditions that has optimal statistical power for measuring HRF shape (Buracas and Boynton, 2002; Liu and Frank, 2004). Scans in both experiments contained an 8 s presentation of a uniform mean field before the first visual stimulus occurred and a 16 s mean field presentation after the final stimulus presentation to allow the HRF to start at and return to baseline.

To further validate the findings of Experiments 1 and 2, we re-analyzed a medium ISI (about 4.5 s) experiment run at 7 Tesla for different purposes (Schumacher et al., 2011). The experiment used a different MR sampling rate (*TR* = 1.5 s) and a different stimulus geometry (four circular patches of contrast-reversing gratings). Results therefor are not directly comparable to Experiments 1 and 2, but are nonetheless informative because they are representative of many common experimental designs. Full details of the data acquisition are published in Schumacher et al. (2011); briefly, T_2_^*^-weighted images (*TE* = 20 ms) were acquired with 2 mm isotropic resolution on a 7 Tesla Siemens scanner while subjects viewed stimuli presented at 5, 10, 30 and 90% contrast. The stimuli were presented in a random ER design, with an average ISI of 4.5 s; each ISI was drawn randomly from the set [3, 4.5, 6 s]. Twelve repetitions of each of the four contrasts were included in each of 5–8 ER scans per scanning session (depending on the subject's stamina); separate block-design localizers during the same scanning session (3 4-min scans) were used to define regions of interest in primary visual cortex for analysis. Data pre-processing and GLM analysis were as described below for Experiments 1 and 2.

### Data acquisition and pre-processing

All MR images were acquired at 3 Tesla using a gradient echo EPI sequence (*TR* = 250 ms, *TE* = 25 ms). Six of the long-ISI datasets and eight of the short-ISI datasets were acquired on a Siemens Trio system with a 12-channel receive-only head RF coil (body transmit); four of the long-ISI datasets and two of the short-ISI datasets were acquired on a Siemens Skyra system with a 16-channel receive-only head RF coil (body transmit). The two different scanners were used for scheduling convenience, and acquisition parameters were matched to the fullest extent possible. *Post-hoc* analyses (not shown) verified that image signal-to-noise ratio, BOLD contrast magnitude, and final results (latency as a function of response amplitude) were not significantly different in the datasets acquired on different scanners. Slices were oriented perpendicular to the calcarine sulcus in the occipital cortex. The field of view was 172 × 144 with a matrix size of 64 × 48 voxels [partial Fourier = 6/8, echo-spacing = 0.46 ms, total read-out time for one image (*T*_RO_) = 16.6 ms], yielding a resolution of 2.7 × 3 × 2.7 mm with four slices total. Flip angle was 40° (except on two long-ISI scans on the Trio for which the flip angle was 80° due to operator error, but SNR was sufficient, i.e., the number of significantly modulated V1 voxels in localizer scans was not decreased, so data were not discarded). Motion compensation was performed on the data from each scan using the MCFLIRT tool provided with FSL (http://fsl.fmrib.ox.ac.uk/fsl). Motion-compensated data were then distortion-compensated using a field map that was acquired during the functional imaging scanning session and the FUGUE toolbox distributed with FSL.

### Region of interest determination

Primary visual cortex (V1) was identified using standard retinotopic mapping techniques (Sereno et al., 1995; DeYoe et al., 1996; Engel et al., 1997) in a separate scanning session. Higher visual areas were not analyzed because they provide weaker responses to full-field checkerboard stimuli, and the analysis of fine timing required particularly high signal to noise ratios. To project retinotopic regions of interest into the current data sets, the mean EPI images from our experimental sessions were aligned (after motion compensation and distortion compensation) to a 3D MP-RAGE volume of the whole brain. Automatic alignment was performed after inversion of voxel intensities to match the T_1_ contrast of the anatomical data (Nestares and Heeger, 2000). Regions of interest (ROIs) comprised voxels within V1 whose coherence (correlation with a sinusoid at the best-fitting phase) exceeded a threshold of 0.3 in the block localizer scans.

Further selection criteria were applied based on mean-normalized variance: for the primary analysis, voxels were sorted by mean-normalized variance (i.e., variance in ER scans after signal was converted to percent signal change) and the top 20% were discarded from the ROI in order to minimize the contribution of large pial vessels to the signal (de Zwart et al., 2005; Olman et al., 2007). Removing this constraint to include all voxels produced qualitatively similar patterns of results with slightly decreased significance for the long ISI data, but increased significance for the short ISI data. Secondary analyses investigated HRF properties as a function of mean-normalized variance, and for this ROIs comprised voxels binned according to mean-normalized variance.

### Data analysis

Data analysis was performed with custom scripts written in MATLAB. First the BOLD signal from each voxel in the ROI was averaged, to create a mean V1 time course. Hemodynamic response functions were estimated from the average V1 time course by deconvolution, using ordinary least squares and a “finite impulse response” model to estimate the BOLD response for 20 s after the stimulus onset for each stimulus condition (Hinrichs et al., 2000; Serences, 2004); Legendre polynomials were used as nuisance regressors to absorb baseline fluctuations with temporal frequencies up to 1/60 Hz. We tested whether the deconvolution could possibly influence estimation of timing, by comparing its results with simple stimulus-locked response averaging for the long ISI data. The two methods yielded identical overall patterns of results, and so we report only the deconvolution results here. It was common for the first 10 s of each scan to exhibit a strong baseline decay; the cause of this was undetermined, but dummy regressors were used in the analysis to avoid contaminating HRF estimates with data from the unstable portions of the scans.

To quantify the timing of each V1 average HRF, we estimated its latency of onset, time to peak (TTP), and full-width-at-half-maximum (FWHM). These parameters are commonly used to describe the temporal characteristics of the HRF (Menon et al., 1998; Thierry et al., 1999; Henson et al., 2002; Liao et al., 2002; Bellgowan et al., 2003; Casanova et al., 2008; Lindquist et al., 2009). Latencies are informative about temporal shifts, whereas FWHM (time between the rise and fall of the HRF) describes whether the HRF shape is more peaked or broad.

**Figure 3:**
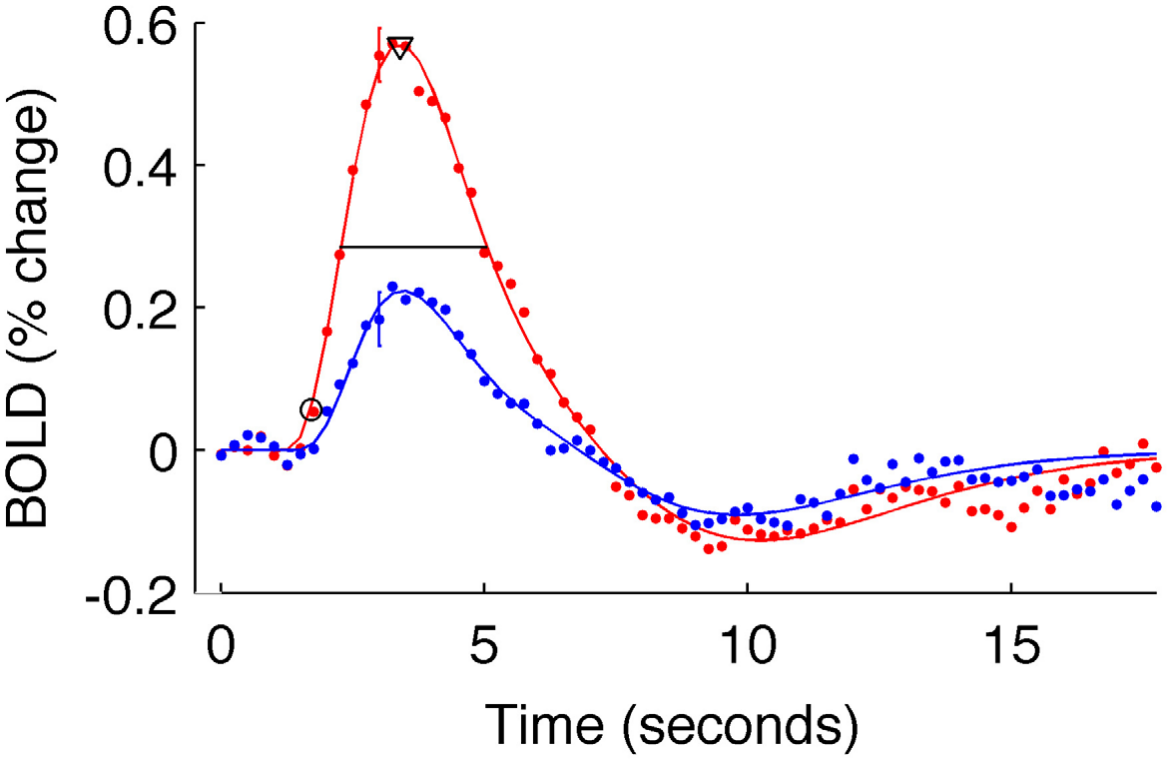
**Estimated HRFs and fit parameters for one typical subject**. Dots are BOLD data for subject 9, averaged within active voxels in V1, for the short-ISI experiment. Color indicates stimulus contrast (blue 5%, red 90%). Error bars show ± one standard error of the mean across voxels for a typical timepoint. Solid line is the fit HRF for these data, used to calculate HRF timing parameters. Onset latency (circle), time to peak (triangle), and FWHM (horizontal line) are illustrated.

To estimate these quantities, each V1 average HRF was fit with a difference of gamma functions model using least squares minimization. Individual HRFs (for each stimulus condition of each subject) for which the fit did not explain at least 80% of the variance were excluded from further analysis (in the primary analysis, two subjects for the 5% contrast condition were discarded from the long-ISI experiment; three subjects' 2.5% contrast responses and one subject's 10% contrast response from the short-ISI experiment). We then estimated onset latency for each V1 average HRF as the time the model HRF first reached 10% of its peak value. To corroborate the trends found at this estimate, we also calculated onset at 50% of the peak response. The same pattern of latencies was observed for both 10 and 50% of the peak value, so all subsequent analyses were performed and reported only for the latencies at 10% of the peak (Figure 3). Time to peak was estimated as the time at which the HRF model reached its maximum amplitude. FWHM was defined by the time from when the HRF rose to 50% its peak until it fell to 50% of its peak. To quantify the amplitude of each HRF we used the value at the peak of the model HRF.

Onset latencies, peak latencies, and FWHM were compared to HRF amplitudes using a linear model to quantify trends. To minimize outlier influence, we used robust regression with an iterative reweighting of the least squares with the bisquare weighting function (Matlab robustfit; Holland and Welsch, 1977; Huber, 1981; Street et al., 1988; DuMouchel and O'Brien, 1989). Fitting in this way proved to be more conservative than the other fitting methods attempted (least squares alone and outlier rejection).

## Results

In Experiment 1, which used long ISIs, higher contrast stimuli produced larger BOLD responses that arose earlier in time. The larger BOLD response for the higher contrast stimuli arose presumably because higher contrast stimuli produce larger responses in V1 neurons. Onset latencies were computed from HRFs fit to the average signal from active voxels in V1 for each subject and contrast condition. These latencies were reliably shorter for the high contrast stimulus condition than for the low contrast condition [*t*_(7)_ = 3.8, *p* < 0.03].

**Figure 4:**
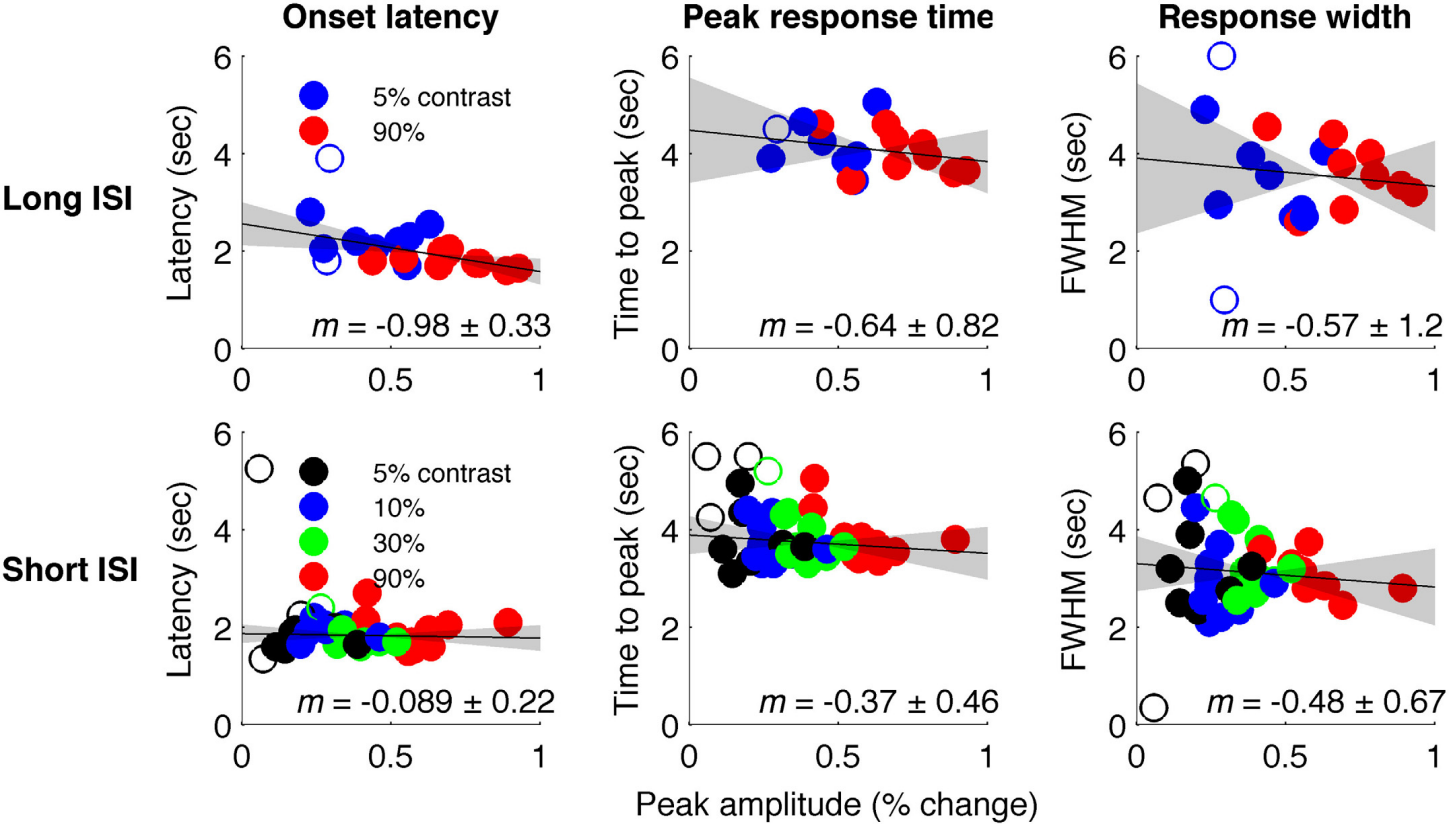
**Timing parameter estimates for individual HRFs. Top:** Long-ISI data. **Bottom:** Short-ISI data. Columns plot (left to right) onset, peak, and FWHM vs. amplitude. Slope (solid line) and 95% confidence interval (gray shading) for robust regression are displayed on each plot. Circles are individual scanning session estimates, with color indicating contrast (black 2.5%, blue 5%, green 25%, red 90%). Empty circles indicate HRFs not included in regression because difference-of-gamma-function fit explained less than 80% of the variance of the HRF.

**Figure 5:**
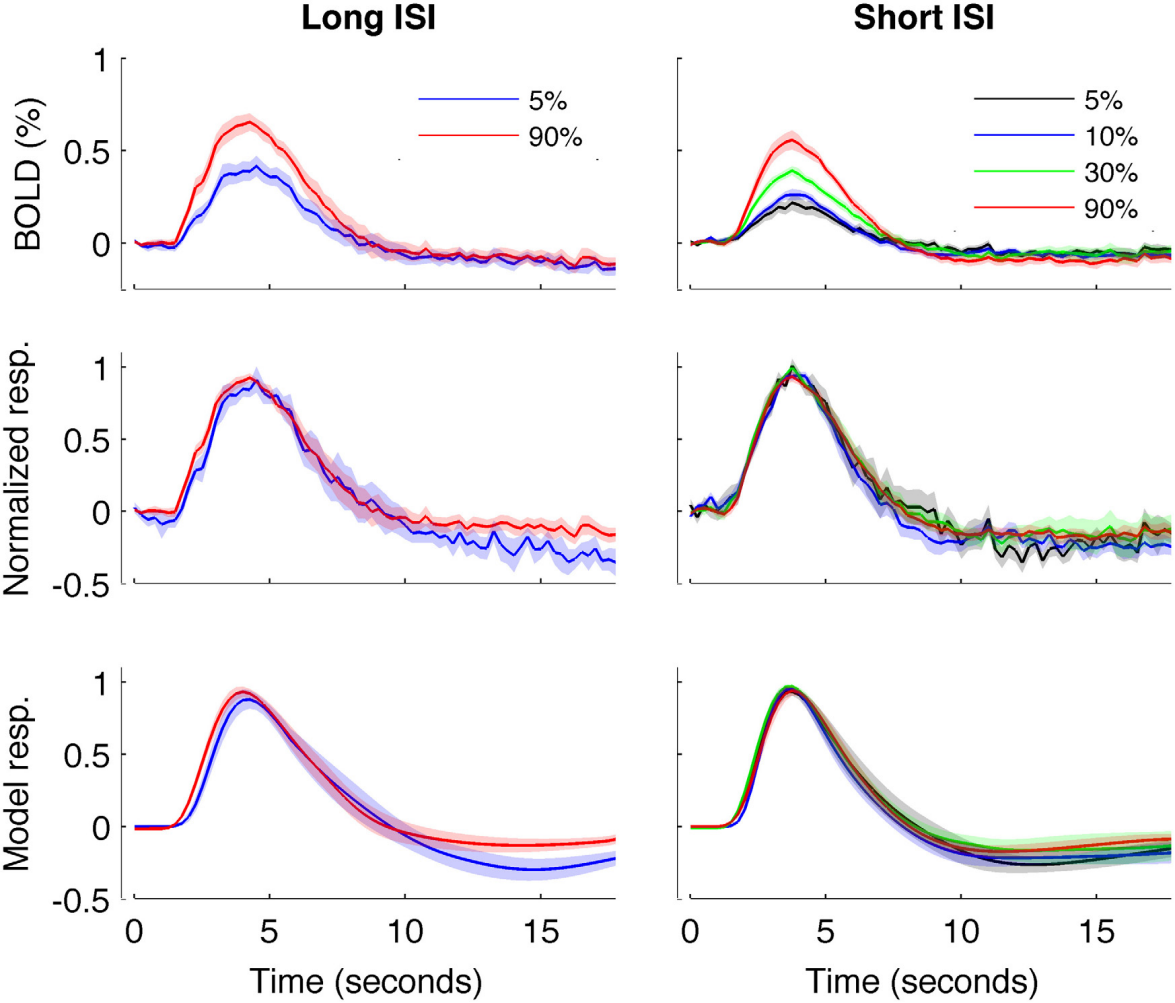
**HRFs averaged across all subjects**. Left: Experiment 1 (long-ISI). **Right**: Experiment 2 (short-ISI). **Top row**: Average HRFs (*n* = 8, 10 for 5 and 90% contrast in long-ISI experiment, *n* = 7, 10, 9, 10 for 2.5, 5, 25, and 90% contrast in short-ISI experiment). **Middle row**: Each HRF scaled by the peak amplitude of fit before averaging, to illustrate shape difference in long-ISI data. **Bottom row**: Average of fits, again scaled by individual estimated amplitude before averaging. Note that, for middle and bottom rows, because of differences in peak timing, average peak value is not 1 even though individual HRFs and fits are scaled to 1 before averaging.

Figure 4 plots the HRF amplitudes (from the average signal of active voxels in V1) as a function of HRF onset latency for each subject and contrast condition. The data show a reliable linear trend for the relationship between response onset and amplitude. Figure 5 shows the average HRFs and fits (scaled to the same amplitude) to qualitatively illustrate the effect. The slope of a linear fit to onset latency as a function of response amplitude for the long-ISI data was −0.98, indicating a decrease of almost 1 s in onset latency for a change in neural activity that produced a 1% increase in the BOLD response.

We repeated our analysis on a second timing parameter, TTP, to compare our results to prior findings, which have observed a TTP that is constant across stimulus contrasts (Lindquist and Wager, 2007; Casanova et al., 2008). Consistent with previous literature, we found no significant correlations between TTP and response amplitude (Figure 4), although a weak trend toward decreasing TTP with increasing amplitude was present. The widths of the HRFs also showed no significant dependence on response amplitude; a weak trend toward decreasing width with increasing amplitude was consistent with the fact that onset shows a stronger dependence on amplitude than TTP.

**Figure 6:**
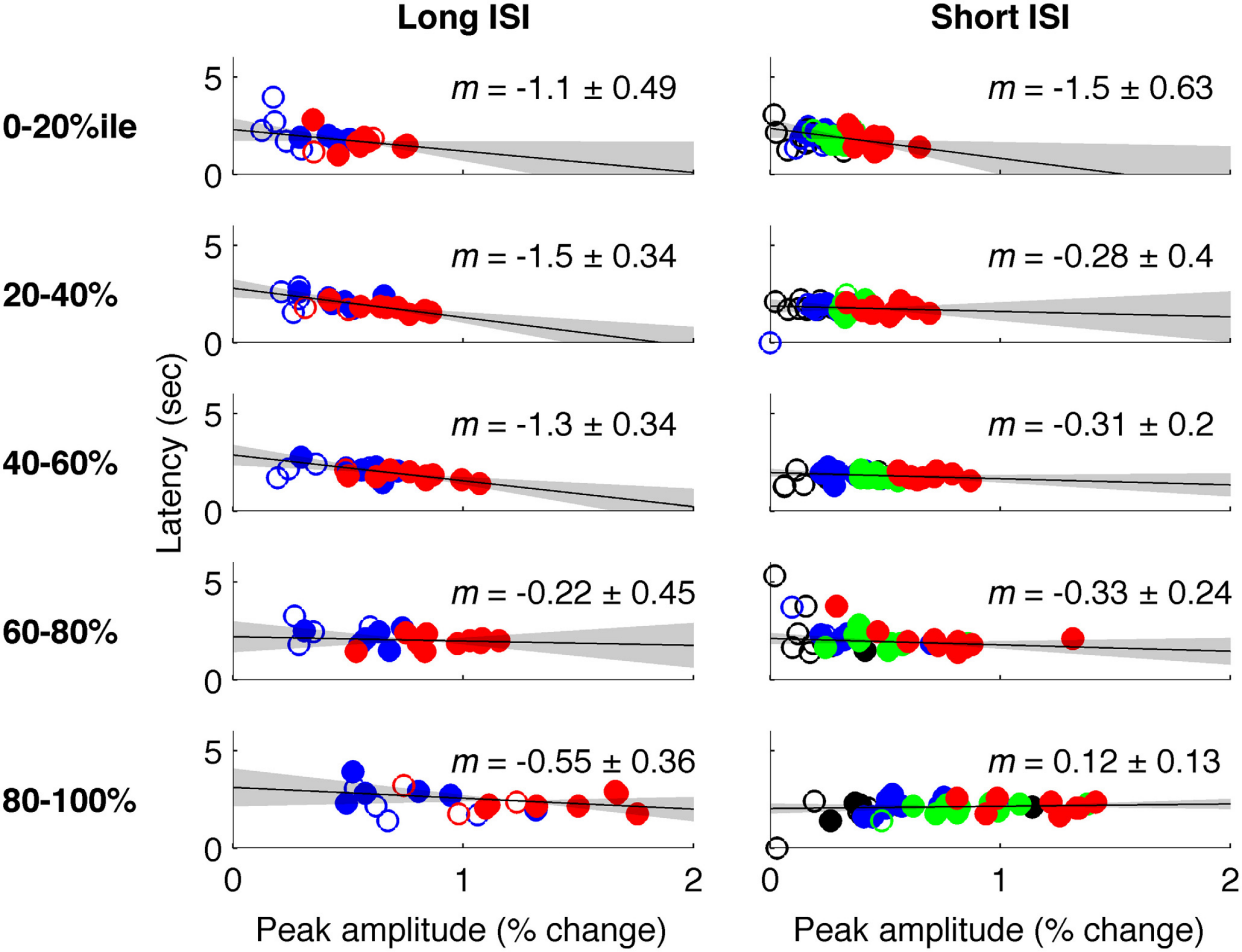
**Latency vs. amplitude when voxels are binned by mean-normalized variance. Left**: Experiment 1 (short-ISI); **Right**: Experiment 2 (long-ISI). Each row represents 20% of voxels, selected according to variance in signal amplitude divided by mean amplitude. **Top row**: The 20% of voxels with lowest mean-normalized variance. Second row: the second quintile of voxels sorted according to mean-normalized variance… **Bottom row**: The 20% of voxels with highest mean-normalized variance. Colors and plotting conventions are as in Figure 4. We use mean-normalized variance as a surrogate for vessel diameter; the strongest relationships between amplitude and latency arise in the voxels with the lowest mean-normalized variance, likely small vessels, and parenchyma.

In Experiment 2, which used very short (1 s) ISI's, no reliable relationship was found between amplitude of neural activity as indexed by the BOLD response, and HRF timing or shape, though trends were in the same direction as in Experiment 1 (Figures 4, 5). However, there is evidence that signal predominated by microvasculature has different temporal dynamics than the general venous signal (Hulvershorn et al., 2005; Kriegeskorte et al., 2010). To test for this possibility, we used voxel variance as a proxy for vessel size; high variance voxels correspond primarily to signal from larger veins and lower variance voxels correspond primarily to smaller veins (Duyn, 1995; Lee et al., 1995; de Zwart et al., 2005; Olman et al., 2007). Voxels were sorted by variance and separated into five bins for both experiments, and for each bin the analysis of onset as a function of amplitude was repeated (Figure 6). In both Experiments 1 and 2, reliable trends relating amplitude and response onset were found for the lowest variance bins, suggesting that observed timing differences are more likely attributable to parenchymal and small vessel signal than to large vessels.

**Figure 7:**
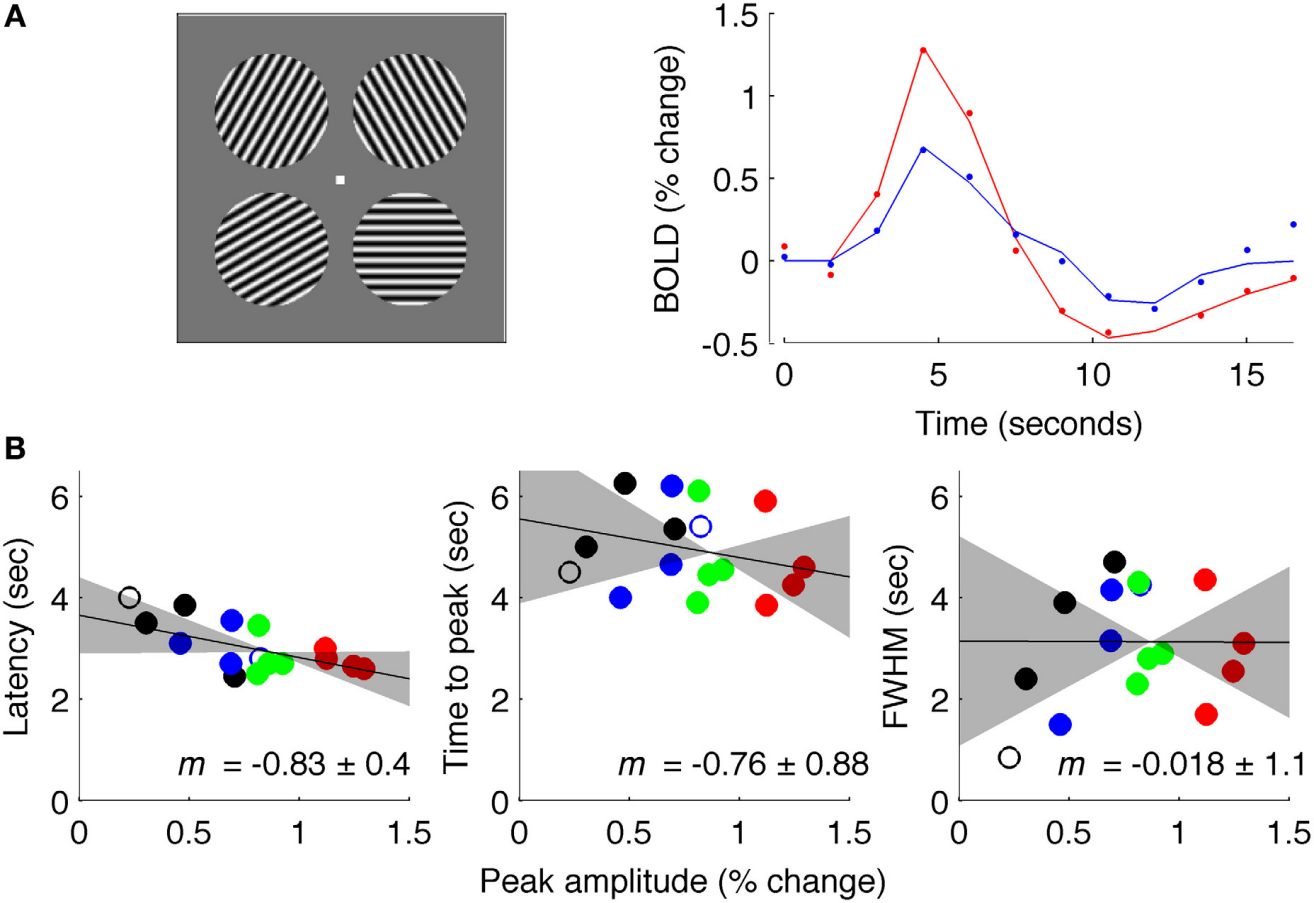
**Results of Experiment 3: intermediate ISI. (A)** Left: stimuli used for experiment at 7 Tesla. Right: estimated fMRI response for 18 s after stimulus onset for representative subject, with solid lines showing fit of difference-of-gamma HRF model. **(B)** Latency, TTP and FWHM for all contrasts (black: 5% contrast; blue: 10%; green: 30%; red: 90%), of all (4) subjects.

Data acquired with an intermediate ISI (4.5 s average, Experiment 3) produced results similar to Experiment 1 (Figure 7). These data were acquired at 7 Tesla for a different experiment (Schumacher et al., 2011) so the signal was sampled with *TR* = 1.5 s instead of *TR* = 0.25 s; additionally, stimuli were circular patches of sinusoidal grating instead of full-field gratings. However, data were analyzed in the same way as for Experiments 1 and 2, and showed a reliable amplitude-dependent onset difference, with a slope of −0.83. These results should be interpreted with caution because of the changes in field strength and TR from data acquired in Experiments 1 and 2.

## Discussion

Our results demonstrate that the timing of the BOLD fMRI signal is dependent upon the amplitude of the neural activity that produces it. When stimuli are reasonably well separated in time, signals produced by stronger neural activity begin earlier in time. The effect of amplitude on timing was large; weak signals were often delayed relative to strong ones by more than a second. This effect was statistically reliable for long (15.5 s) and intermediate (4.5 s) ISIs, but for very short (1 s) ISIs trends in the same direction were only reliable in the lowest variance voxels (parenchymal rather than large-vein signal).

Our results are also consistent with two sets of prior measurements, neither of which commented upon observable (though perhaps not statistically reliable) decreased BOLD onset latencies for larger neural responses. Grinband et al. (2008) measured BOLD responses to flickering checkerboards presented at 5, 10, 20 and 40% contrast, with an average ISI of about 6 s. Their data show decreasing onset latencies as a function of response amplitude on the order of 1 s over the range of BOLD response amplitudes measured. Wan et al. (2006) measured responses to gratings that ranged from 1 to 100% contrast with an ISI of 25 s. Their data show a similar pattern, with delays decreasing by about 500 ms per 1% change in BOLD signal. Notably, this paper also measured neural response with electrophysiology; the EEG data show large changes in visual evoked potential amplitude, but only a 50 ms change in onset time over the contrast range tested.

Thus, BOLD responses produced by larger amounts of neural activity arise more rapidly. While the present data do not identify the causes of this effect, our results do rule out several possibilities. First, the effects measured here are too large to be accounted for by changes in neural latency that arise as a function of response strength. Neural responses measured with single unit recording show changes in response onset times of less than 100 ms as response strength varies over a large range (produced by increasing stimulus contrast from 5 to 78% Gawne et al., 1996).

The fine timing of fMRI responses can also depend upon the presence of preceding responses that are relatively close in time (McClure et al., 2005; Zhang et al., 2008; de Zwart et al., 2009). However, the fact that the long-ISI experiment showed a stronger onset effect than the short-ISI experiment indicates that temporal precedence is not to blame for the onset effects measured here.

The delay for smaller amplitudes is also not likely to be due to signals arising from large vessels. Large veins are marked by high variance and are known to have longer response latencies (de Zwart et al., 2005; Hulvershorn et al., 2005); low-variance voxels are typically dominated by small-vein signal, arising in the capillary bed and penetrating intracortical venuoles and referred to most commonly as parenchymal signal. (The utility of variance as a surrogate for vessel diameter was confirmed by the fact that the average response amplitude in the high-variance bins was typically twice that of the amplitude in the low-variance bins.) Because we observed the strongest onset changes in the voxels with the lowest mean-normalized variance, the onset differences likely arise in the parenchyma and not in large veins.

The effects of amplitude on onset appear to depend upon ISI. Experiment 1, with a long ISI showed reliable effects, while Experiment 2, with an extremely rapid 1 s ISI showed only numerical trends for the relationship. Experiment 3 used an intermediate ISI: the onset differences were evident even with slow sampling and were similar in magnitude to Experiment 1. Thus, ER experiments with commonly used ISIs (in the 4–6 s range) can expect that larger neural responses will have more rapid BOLD signals.

Why did Experiment 2 show mainly unreliable trends? One possibility is that the BOLD signal change was weaker; mean amplitudes in Experiments 1 and 3 were over 0.5% signal change, but were much lower in Experiment 2. This could indicate either that the amplitude-onset relationship is weaker for lower amplitudes, or that Experiment 2 simply lacked the statistical power to detect the relationship due to low SNR. Another, perhaps more interesting possibility, is that the amplitude-onset relationship depends upon hemodynamics starting at or near resting state levels. Note, however, that Experiment 3 shows that amplitude-dependent onset differences can be evident even when hemodynamic responses overlap significantly. Glover (1999), has shown that deconvolution models are effective down to an ISI of 4 s, but not below, so 4 s may represent a point at which neurohemodynamic coupling changes meaningfully, which could explain why Experiments 1 (20 s ISI) and 3 (4.5 s ISI) but not 2 (1 s ISI) showed amplitude-dependent onset differences.

Our data are inconclusive about whether the shift in HRF onset is due to either a shift of the entire response earlier in time or just a shift of the onset. The former model would predict a shift in the peak latency in addition to the shift in onset, while the latter would predict an increase in FWHM. Neither of these was seen with statistical significance, though there were numerical trends for both.

One concern in any study aiming to characterize the speed of the HRF is the determination of onset time, i.e., the departure of signal from the baseline response. The use of rapid sampling in the 3T data is one strength of the present study for obtaining reliable estimates of the timing properties of the contrast evoked HRF. Undersampling is known to misrepresent the fine timing characteristics of the HRF by introducing up to a 50 ms onset lag, over-representing the large vascular response to the stimulus, and creating up to a 100 ms shift in the peak time (Dilharreguy et al., 2003). However, rapid sampling reduces SNR by approximately 40% (250 ms TR compared to 1500 ms TR), which makes it difficult to estimate a true baseline from which to measure HRF onset. To overcome this difficulty, we fit the HRFs with gamma functions rather than estimating baseline and onset more directly from our data.

The generality of these results remains to be fully explored. While similar, but uncommented upon, results have already been reported from other labs (Wan et al., 2006; Grinband et al., 2008), it is unknown whether the same relationship between neural activity and HRF onset will exist for parameters that modulate neural activity other than visual contrast. There is no a *priori* reason to doubt that this would be the case, however. Whether neural activity in other parts of the brain shows the relationship also remains to be tested. The timing of HRFs from subcortical visual regions are already known to differ from each other and from those in cortex (Lau et al., 2011; Yen et al., 2011) making this a promising area for future research.

BOLD onset differences between low and high response amplitude neural responses have three important implications for the analysis of fMRI data. First, and perhaps most obviously, our results bear upon studies that attempt to use fMRI to make inferences about the relative timing of various neural processes, even within the same cortical region: processes that generate only low amplitude neural and fMRI responses will likely have their onsets systematically underestimated compared to processes that generate larger responses.

Second, timing differences will lead to systematic error in estimation of response amplitudes if analyses assume a fixed HRF (McClure et al., 2005), as is the case in many widely-used implementations of the general linear model (GLM). If, for example, a canonical HRF is derived from high amplitude responses, then it will fit low amplitude signals less well, which will lead to underestimation of their response amplitude. Our results thus support the inclusion of temporal derivatives, or other methods to allow temporal shifting across conditions, into GLM analyses.

Finally, our results also have implications for some modeling of causal relationships between regions of neural activity. Timing is one factor that can be used in such analyses, since causes must arise before effects. Our results suggest that larger neural effects generate fMRI responses that arise generally earlier than weaker effects; unless care is taken to remove this amplitude confound, analyses may spuriously infer that larger effects are causes of smaller ones.

### Conflict of interest statement

The authors declare that the research was conducted in the absence of any commercial or financial relationships that could be construed as a potential conflict of interest.
